# Feasibility of Roux-en-Y Gastric Bypass with the novel robotic platform HUGO™ RAS

**DOI:** 10.3389/fsurg.2023.1181790

**Published:** 2023-06-05

**Authors:** Marco Raffaelli, Nikolaos Voloudakis, Francesco Pennestrì, Pierpaolo Gallucci, Cristina Modesti, Giulia Salvi, Francesco Greco, Luigi Ciccoritti

**Affiliations:** ^1^ U.O.C. Chirurgia Endocrina E Metabolica, Centro Dipartimentale Di Chirurgia Endocrina E Dell'Obesità, Fondazione Policlinico Universitario Agostino Gemelli IRCCS, Rome, Italy; ^2^ Centro di Ricerca in Chirurgia Delle Ghiandole Endocrine e Dell'Obesità, Università Cattolica del Sacro Cuore, Rome, Italy; ^3^ U.O.C. Anestesie Delle Chirurgie Generali e dei Trapianti, Fondazione Policlinico Universitario Agostino Gemelli IRCCS, Rome, Italy; ^4^ Dipartimento di Scienze Biotecnologiche di Base, Cliniche Intensivologiche e Perioperatorie, Università Cattolica del Sacro Cuore, Rome, Italy

**Keywords:** HUGO™ RAS, Roux-en-Y Gastric Bypass, robotic surgery, minimal invasive surgery, RYGB, bariatric surgery, docking setup, complications

## Abstract

**Introduction:**

Robotic assisted surgery is a rapidly developing field of minimally invasive bariatric surgery in the last 20 years. Its wide diffusion has led to the development and standardization of robotic assisted approaches for bariatric operations. In this study, we present the first four Roux-en-Y Gastric Bypass (RYGB) operations performed with the new Hugo™ RAS system (Medtronic, Minneapolis, MN, USA).

**Methods:**

In January and February 2023, 4 consecutive patients scheduled for minimal invasive Roux-en-Y-Bypass were selected and underwent the procedure robotic-assisted with the new platform. No exclusion criteria were applied.

**Results:**

Four patients, two females and two males, underwent RYGB with a median BMI of 40 Kg/m^2^ (range: 36–46) and diabetes mellitus in two cases. The median docking time was 8 min (range: 7–8.5) and the median console time was 127.5 min (range: 95–150). A description of the operating theatre, robotic arms and docking setup is provided. Procedures were performed without intraoperative complications and no conversion to laparoscopy or open surgery was noted. No additional ports were needed to be placed. System's function and docking were uneventful. No early post-operative complications were observed.

**Conclusions:**

Based on our initial experience, RYGB with the Hugo™ RAS system is feasible. This study provides the configurations necessary to perform RYGB with the Hugo™ RAS system as well as general information and insights from our preliminary experience.

## Introduction

As the prevalence of obesity is projected to rise in the immediate future, reaching over 20% in more than half of European countries by 2025, the demand for bariatric operations is expected to follow suit (1). Roux-en-Y Gastric Bypass (RYGB) has been the most commonly performed bariatric operation in Europe and is among the most popular in the world, particularly for coexisting gastroesophageal reflux disease (2).

The gold standard laparoscopic technique is well documented and established, yielding superior results to the previous open approach (3). However, bariatric patients, and especially the super obese subgroup, present with technical challenges in minimal invasive surgery, due to the thick abdominal wall, enlarged liver, and increased visceral fat. Consequently, the working space and exposure is limited, while torque forces are amplified (4, 5). In addition, the further preference for hand-sewn anastomosis construction, has steered several bariatric surgeons towards the more ergonomic robotic solutions (6, 7).

The first robotic-assisted bariatric procedure was performed in 1998 by Cadiere for gastric banding (8), soon followed by a robotic-assisted RYGB (RRYGB) (9). Since then, proportions of RRYGB operations performed, utilizing the Da Vinci platform (Intuitive Surgical, Sunnyvale, CA, USA), have steadily increased, reaching a percentage of 16.7 by 2020 in the U.S. (7). RRYGB has been associated with lower rates of complications but longer operative times and higher costs when compared to laparoscopic RYGB (LRYGB) in meta-analyses (3, 10, 11). However, studies included had great heterogeneity in surgical techniques applied, surgeons’ expertise in robotic operations, and a retrospective design.

Wider diffusion of robotic assisted bariatric operations has been limited thus far due to platform availability and cost-related concerns. Novel platforms for robotic assisted surgery are recently emerging, promising new features and competing for a place in the market, which might mitigate the current overcost and accessibility obstacles. Among new platforms, a recently introduced system is the Hugo™ (Medtronic, Minneapolis, MN, USA). The Hugo™ robotic assisted surgery system (RAS) was introduced in the European market in March 2022, having first received CE (Conformité Européenne) approval for gynaecological and urological procedures, while recently this approval was extended to general surgery (October 2022).

The Hugo™ platform consists of a system tower, an open console and four individual arm-carts. Each cart may move independently, facilitating various placements and limiting the risk of collision. The robotic arms have six joints, promising a wider maneuver range. The surgeon is seated on an open console with a 32 inch-widescreen HD-3D display and dedicated glasses. The handgrips design simulate a “pistol grip” and the footswitch panel contains controls for the camera, energy sources, and the reserve arm.

The safety and feasibility of the Hugo™ platform has already been tested in urological (12–16), gynecological (17, 18) and adrenal procedures (19). The aim of this report was to assess its feasibility and provide technical details of the setup in multi-quadrant operations such as the RYGB.

## Methods

Between late January and early February 2023, four consecutive informed patients underwent RRYGB with the Hugo™ RAS system in our Institution, a tertiary referral center for bariatric surgery. No specific exclusion criteria were applied for patient selection, apart from being scheduled for minimal invasive RYGB. Operations were performed by a surgeon experienced in both laparoscopic and robotic bariatric operations (MR). Participating surgeons and nurses had previously completed the technical training on Hugo™ RAS System delivered by Medtronic at the ORSI Academy, Aalst, Belgium and were familiar with the platform from performing other types of operations with the same system (19). Informed consent of all participating patients was acquired.

### Surgical technique

#### Patient position and trocar placement

Under general anesthesia, the patients were placed in a supine position with the legs split (“French” position). Pressure points and bony prominences were padded for protection. Patients’ body was secured with a gel pad and strapped across the thighs to avoid any shifting in the reverse Trendelenburg position (20°).

**Figure 1:**
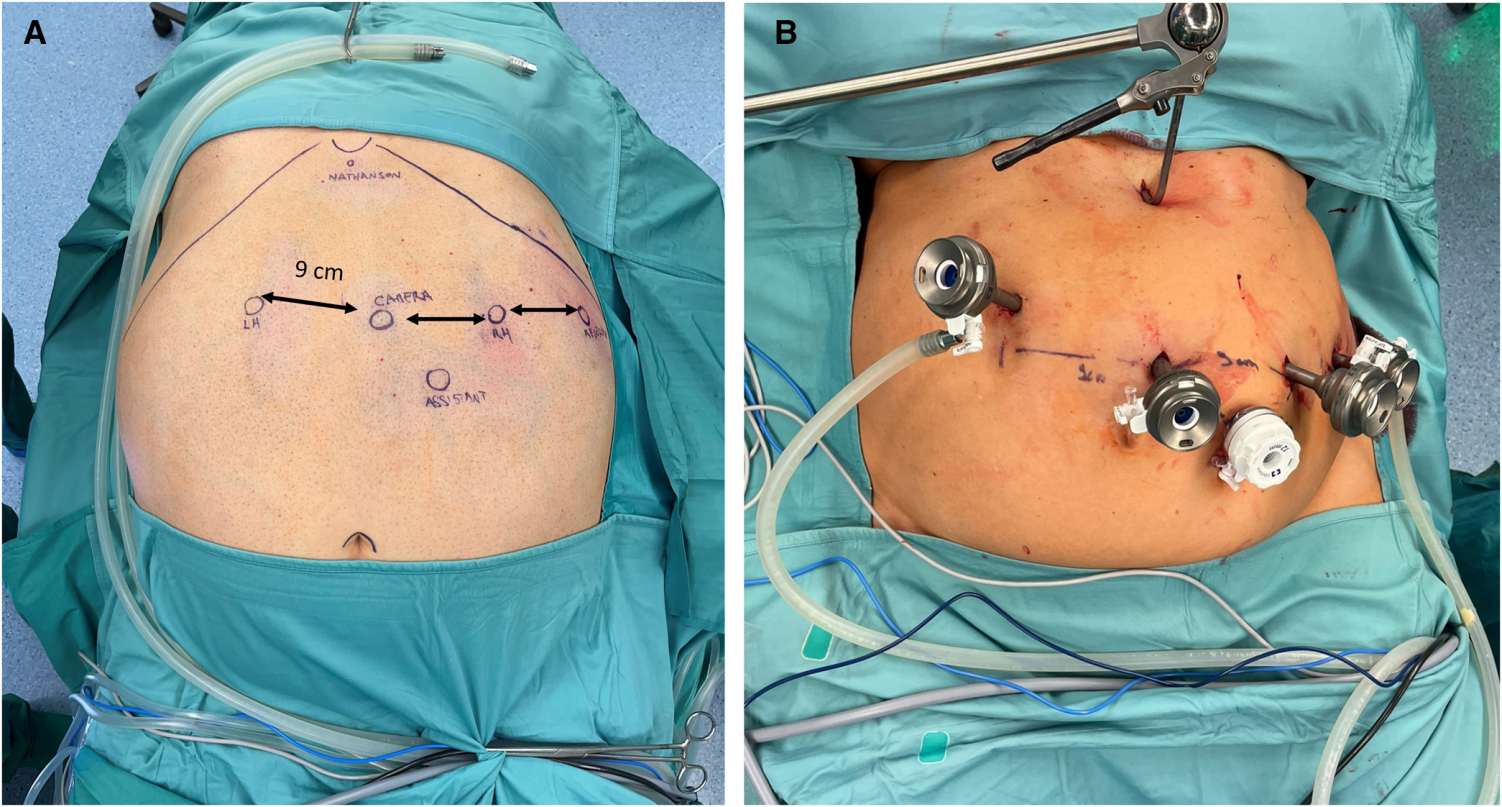
(**A**) Trocars position for RYGB with the Hugo™ RAS system in patient No 3. Camera, endoscope; RH, surgeon's right hand; LH, surgeon's left hand; Nathanson, liver retractor; Assistant; Reserve. (**B**) Following trocar and Nathanson retractor placement in patient No. 2.

Caution is advised when placing the robotic ports. A minimum distance of 8 cm between them is required to avoid collisions during the operation. The first port, a 11 mm camera port, was placed supraumbilically, slightly on the left at approximately 15 cm below the xiphoid process. Following pneumoperitoneum establishment, three 8 mm robotic trocars were then inserted, one on each flank and one in the left subcostal space, along a parallel line (Figure 1). A 12 mm accessory trocar, to be used by the assistant, was placed inferiorly, in the middle of the distance between the camera trocar and the surgeon's right-hand trocar. Ultimately, a Nathanson's liver retractor was inserted in the subxiphoid area.

#### Docking

**Figure 2:**
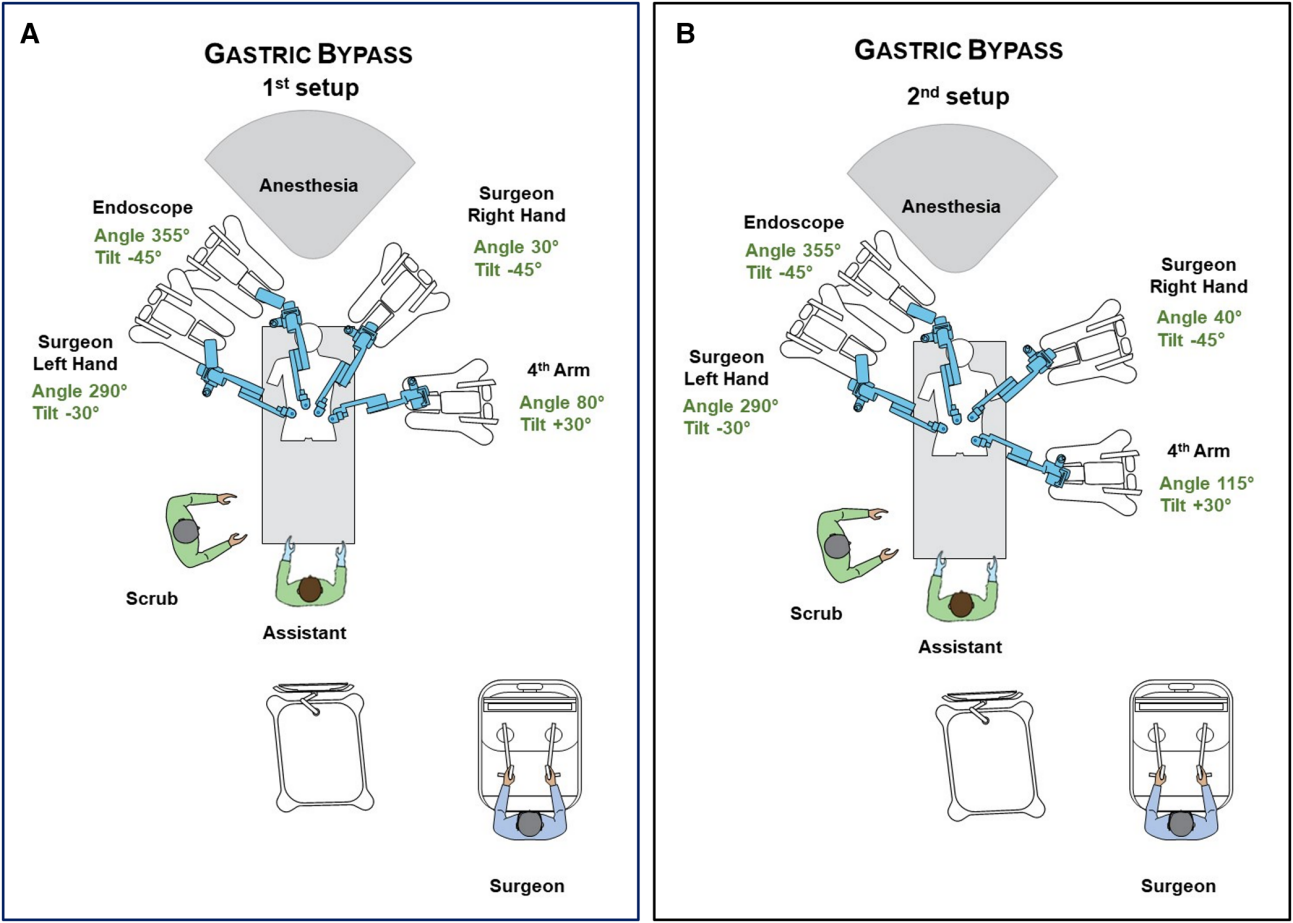
(**A**) Operative room settings, positions of platform's components and surgical team members during RYGB. Description of arms docking and tilt angles. (**B**) Modifications after first operation. Description of arms docking and tilt angles. Further small changes can be performed to optimally match patient's body type.

The Hugo™ system consists of 4 independent arm carts. Each arm requires its own settings, that can be adjusted depending on the patient's body type. Two main settings are required to configure each arm. One is the *tilt angle*, which is the vertical angle of the arm in respect to the flat operative bed (0°) and can be adjusted by lifting upwards or downwards the arm's nose. The other is the *docking angle*, which is the clockwise horizontal angle between the head of the patient (0°) and the arm's direction (19). Configurations were defined by our team along with the company's personnel prior to the operations on a surgical manikin (Figure 2). Adjustments in the settings of the third and fourth robotic arms were made in the second patient to match his specific body type. Those adjustments also created more room for the anaesthesia personnel and prevented collisions (Figure 2). In all operations, a bipolar fenestrated grasper was used for the left surgeon's hand, a monopolar curved sears (with protective tip cover) for the right, switched with a large needle-driver during the anastomosis construction. A secure Cadiere forcep or a double fenestrated grasper was used for the fourth robotic trocar.

**Figure 3:**
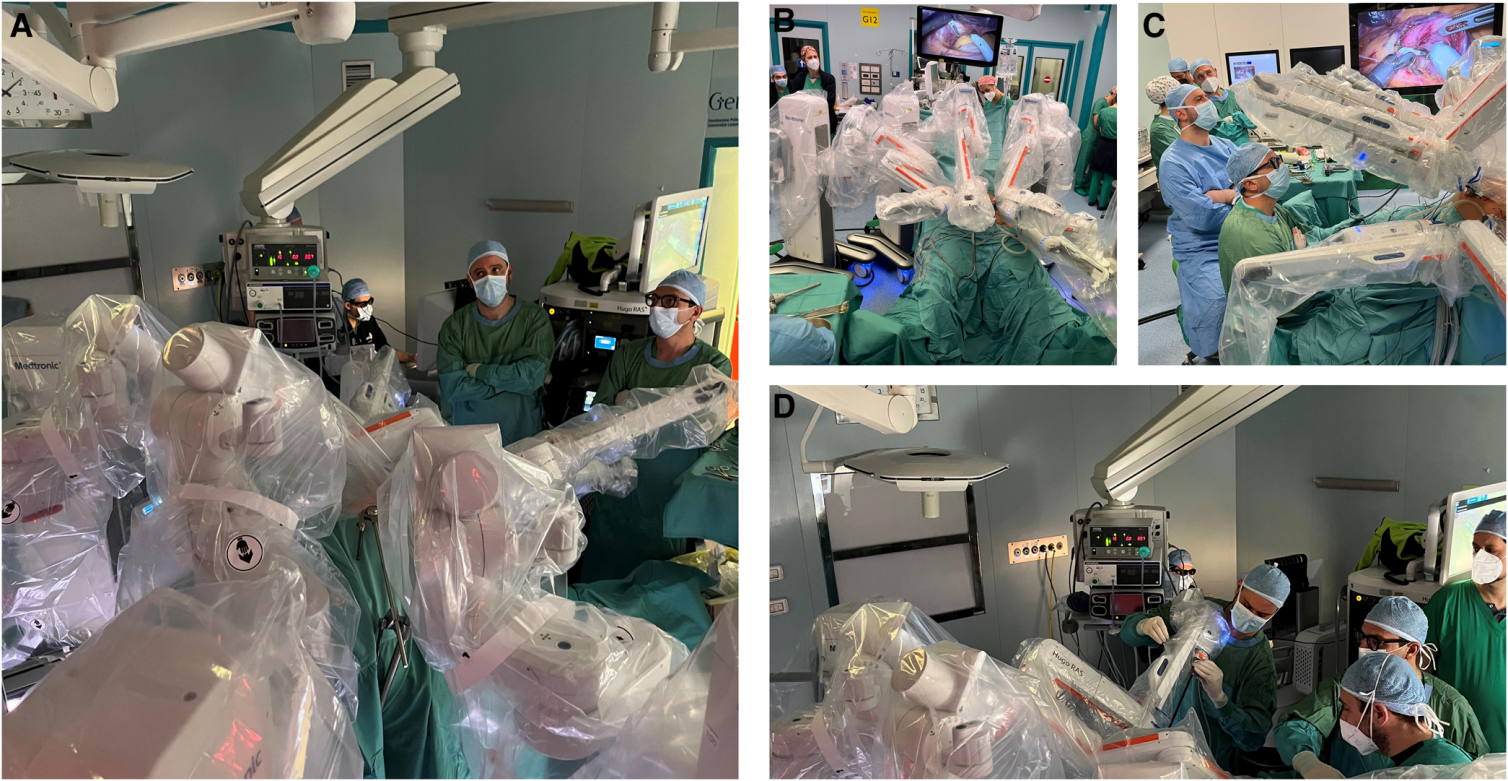
(**A**) Intraoperative image during the docking. (**B**) Intraoperative image exhibiting the anesthesiologist's position. (**C**) Intraoperative image exhibiting the assistant's position. (**D**) Intraoperative image exhibiting the console position during surgery.

Figure 3 includes operating room pictures during the procedures. It should be noted that, differently to the Da Vinci Xi™ system, the platform does not have a “memory” of the docking for each procedure and has to be manually configured separately each time.

#### Operation

We applied the antecolic double-loop technique for the RYGB (20). The first step of RRYGB was the creation of the gastric pouch. Following adequate exposure of the gastroesophageal junction, by retracting the left liver lobe with the Nathanson retractor, the lesser sac was entered along the lesser curvature approximately 6 cm from the oesophago-gastric junction and the stomach. The bed-assistant introduced the laparoscopic linear stapler (Signia™ stapling system, Medtronic, Minneapolis, MN, USA) through the accessory trocar. The gastric pouch was created using three purple cartridges (one horizontal, two vertical): a 36 F orogastric bougie was used for calibrations. The trocars’ setup that has been described for the robotic procedures is clearly derived from the one we use for laparoscopic RYGBs. More in details, three years ago we shifted the trocars’ configuration from the one most reported in literature to one with all but one 5 mm trocars, in order to reduce the incidence of trocars’ hernias. So, we utilized only one 12 mm trocar (for the stapler device) that is placed on the patient's left side. Obviously, this imply a rotation of the laparoscopic stapler nearly to 90’, that is possible thanks to the Signia™ stapling system (Medtronic, Minneapolis, MN, USA). With this technique, during these 3 years (about 400 RYGBs/year) we have not experienced any technical difficulties or lengthening of operating times. A small bowel loop approximately 75 cm distally was selected and brought upward in an antecolic fashion without tension. A robotic, hand-sewn, end-to-side gastrojejunal (GJ) anastomosis was performed with two layers running suture (Stratafix™ and PDS™) (21). A second loop of small bowel, 150 cm from the GJ anastomosis alimentary side, was identified and brought up to perform an entero-enteric side-to-side stapled anastomosis (single bronze cartridge) between the second loop and afferent limb of the GJ anastomosis. The insertion holes were then closed with a running absorbable suture (Stratafix™). The integrity of the two anastomosis was verified with a blue methylene and pneumatic test through a nasogastric tube positioned in the efferent limb. A gauze placed on the GJ anastomosis was then checked by the surgical staff for presence of methylene blue. The last step was the creation of the Roux-en-Y, by dividing the jejunum between the two anastomoses via a linear stapler (single bronze cartridge, Signia™). A drain was placed posteriorly to the GJ anastomosis. Although there are conflicting opinions in literature regarding the use of drainage (particularly in ERAS/fast track protocols) we use it routinely in clinical practice, without any difficulties in early patient mobilisation (4 h after the end of the surgical procedure) or increase in surgical site infections.

#### Post-operative protocol

A standard postoperative protocol, personalized for bariatric patients was used. All patients remained nil per os until an upper gastrointestinal (UGI) contrast (Gastrografn®, Bracco SpA, Milan, Italy) study was performed on the first post-operative day (22). Liquid diet commenced after the UGI contrast study, if no leak was observed, and clinical course was uneventful. Routine complete blood examination and blood count were obtained on 1st post-operative day in all patients. The severity of postoperative complications was rated according to the Clavien–Dindo classification (23). Patients were discharged 24 h after the surgical procedure if the following conditions were met: no clinical complications or postoperative biochemical and imaging alterations occurred; oral alimentation was tolerated; autonomous in life activities; the discharge was accepted by the patient. The complete post-operative and follow-up protocol has been previously described in detail and is beyond the scope of this study (24, 25).

## Results

Two female and two male patients underwent RRYGB with the Hugo™ RAS platform. Patients’ characteristics, operative details, and post-operative course are shown in Table 1. There were no intraoperative complications or system failures. All operations were completed without additional port placement or conversion to either laparoscopic or open surgery. The console times, in chronological order, were 150, 135, 120 and 95 min respectively, while the total operative times 190, 180, 150 and 130 min respectively. The reduction of the time required between cases was evident, potentially signifying that the surgical team's adaptation to the new platform requires a very limited number of cases, if previous familiar with RRYGB and the new platform. The length of hospital stay ranged between one and two days in all cases (Table 1).

**Table 1:** Patients’ characteristics, operative data, and post-operative course.

	Patient 1	Patient 2	Patient 3	Patient 4
Age (years)	48	52	53	41
Sex (Male/Female)	Female	Female	Male	Male
Weight (kg)	121	97	131	101
Height (cm)	163	156	180	167
Body Mass Index (Kg/m^2^)	46	40	40	36
Comorbidities	–	–	Diabetes	Diabetes
Previous Abdominal Surgery	Yes, open	Yes, open	Yes, laparoscopic	No
Docking time (min)	8.5	8.5	7	7.5
Robotic arms used	4	4	4	4
Console time (min)	150	135	120	95
Total operative time (min)	190	180	150	130
Arm collision instances	2	0	0	0
Intra-operative complications (Yes/No)	No	No	No	No
Post-operative complications (Yes/No)	No	No	No	No
Length of hospital stay (days)	1	2	1	2

In the first operation, there were a few instances of clashing between the robotic arms extra-abdominally, namely between arm 3 and 4. This did not lead to any noteworthy time delay or adverse events, since there is a built-in alarm system that momentarily stops the instruments until the operator unblocks them manually. To avoid such occasions, the surgical team has to first ensure that the distance of the robotic trocars is at least eight centimetres. Furthermore, small adjustments in carts placement, docking and tilt angles of the arms had to be made as shown in Figure 2A and Figure 2B, in order to provide more ample space for each arm extra-corporeally. Lastly, abrupt manoeuvres should be avoided. By applying those modifications, this issue was resolved in the following patients.

## Discussion

In this study, we demonstrated the feasibility of RRYGB with the Hugo™ RAS and described the platform’s configurations applied.

Since our initial experience with the Hugo™ RAS platform in performing adrenalectomy (19), the next logical step was to assess the performance of the system in multi-quadrant operations that also require more advanced minimal invasive skills, such as the construction of hand-sewn intestinal anastomosis.

In our centre, since performing the first robotic bariatric procedure in 2013, we have performed more than 4,177 bariatric procedures in total, but only approximately 90 cases (≈2%) were scheduled for robotic approach, half of them being RYGB and the remaining single-anastomosis duodenal switch operations (SADI-S, single anastomosis duodeno-ileal bypass with sleeve gastrectomy) (24, 25). The main limitations in further application being robotic operative room availability and economic concerns, forcing us to conserve the robotic-approach predominantly for more challenging procedures and/or patients (super-obese, revisional surgery, RYGB, SADI-S) (24, 26). Thus, the opportunity to introduce the Hugo™ RAS platform, in an already established robotic bariatric program, was met with guarded enthusiasm in our Institution.

Although the two platforms are different in design and handling, robotic skills learned and daily applied feel transferable so far. Recent published evidence on simulation trainers, seem to validate our “intangible” experience, signifying that in large medical centres, robotic platform exclusivity is far from mandatory (27).

The outcomes of the first four patients that underwent RYGB with the new robotic platform were promising with docking times ranging from seven to eight and a half minutes and console time ranging from 95 to 150 min with a descending trend. No complications were noted either intra- or post-operatively and patients were discharged one or two days postoperatively.

Although the number of cases were limited, a few preliminary observations could be made that might prove useful to other early adapters. Hugo™ RAS has certain distinct differences from its competition due to the four separate cart-arms. Each arm has a dedicated cart and can move independently. In operations not requiring a fourth arm, the fourth cart can be moved outside of the operating room to save vital space. The separate arms design allows for a great variety of modifications during setup and individual arm settings. By adjusting the carts’ position, docking and tilt angles, the surgical team was able to better match the patient's body type and operation demands. However, in contrast to the Da Vinci Xi platform, the settings must be set manually in each operation. This is often inconvenient, since in the Da Vinci Xi system, following endoscope insertion and pointing towards the desired anatomical point, the system automatically optimises the configurations. Another point that early adapters should note is the length of the robotic arms of the Hugo™ system, which predispose for collisions both between the robotic arms, and between the arms and the assistant. After the first case, adjustments were made and presented here to address this issue and provide more ample space for the personnel involved. The system appears more portable than the Da Vinci Xi since the carts can be moved separately between operating rooms easier. This might prove advantageous in centres lacking a dedicated robotic operating room. In addition, the open console design enabled the surgeon to sit in an upright position, while better keeping in touch with his surroundings. Meanwhile, multiple observers were able to “share” the same screen, provided they wore the specially designed glasses. With the Da Vinci console this was only possible via a second console, if available.

Concerning optimal room utilization, the application of a liver retractor on an epigastric position was essential to avoid the presence of a second surgical assistant. This allows for adequate space when positioning the arm carts. In our experience with both platforms, the Nathanson liver retractor is safe, even in time-consuming operations, and no transient liver dysfunction has been noted thus far. As it has been suggested in previous publications, traction forces imposed are low and we recommend its use especially in RRYGB performed with the Hugo™ RAS (28–31).

It should be also noted that, thus far, indocyanine green (ICG) angiography is not supported by the Hugo™ RAS system. ICG angiography can be useful in cases of doubtful perfusion of the gastric pouch and anastomoses, especially in revisional surgery, where the technical advantages of the robotic platform can be decisive, but thus far the Hugo™ RAS system does not support it, which is a temporary limitation of the platform (32–34). Another limitation of the Hugo™ platform is the lack of robotic staplers that would allow to complete a totally robotic procedure. However, while waiting for similar devices, it was possible to safely accomplish the procedure using a conventional laparoscopic linear stapler introduced by the bed-assistant through the accessory 12 mm trocar. We recommend the use of long-shaft laparoscopic staplers, taking into account the more caudal position of the assistant's trocar. Similarly, the lack of robotic vessel sealing systems, was overcome with use of a combination of mono- (scissors) and bipolar cautery systems. Conversely, the lack of vessel sealing system is a limitation to apply this platform for more complex operations (i.e., SADI-S, duodenal switch). Performing the gastro-jejunal anastomosis manually has been associated with favourable outcomes in individual publications, but not systematically proven superior (35–37). As, in our perspective, it is one of the main benefits of robotic assisted surgery, the performance of the Hugo™ system in this regard was comparable, if not slightly superior during the manual suturing, to the Da Vinci platform. However, due to the limited number of cases, we were unsure if this was only subjectively perceived, or might be justified by a more sharp and detailed vision, or a more ergonomic sitting position of the operator, ultimately improving hand-to-eye coordination.

On the other hand, the efficiency of certain instruments turned out to be lower, having observed a poor grasping capacity of the Cadiere forceps and slightly reduced delicacy of the bipolar fenestrated grasper, raising some concerns during the manipulation of the intestinal loops. Furthermore, the robotic instruments in Hugo™ are slightly shorter than the ones of the Da Vinci Xi system (about 3 cm) demanding an extremely accurate positioning of the trocars. Indeed, while a too cranial (high) positioning facilitated the preparation of the gastro-entero-anastomosis, it rendered the execution of the entero-entero-anastomosis more cumbersome and vice versa in cases of too caudal positioning of the trocars. This reduced length might prove problematic in general during multi-quadrant operations and might need to be addressed in the future.

Another consideration should be the difference between the Da Vinci™ endoscope and that of the Hugo™ RAS. As described, an 11 mm trocar is mandatory for the Hugo™ endoscope to be docked, while in the Da Vinci™ system, the endoscope can be placed in any of the 8 mm trocars. This could be time consuming in operations demanding switching of the robotic instruments’ placement.

In conclusion, the Hugo™ Robotic Assisted Surgery system is a promising platform for performing operations involving advanced minimal invasive skills, such as anastomosis construction. In this initial experience, we demonstrated its safety and feasibility in performing RYGB. Nonetheless, larges series are needed to validate our initial impressions and draw definitive conclusions.

## Data Availability

The raw data supporting the conclusions of this article will be made available by the authors, without undue reservation.

## References

[1] Pineda E Sanchez-Romero LM Brown M Jaccard A Jewell J Galea G Forecasting future trends in obesity across Europe: the value of improving surveillance. Obes Facts. (2018) 11:360–71. 10.1159/000492115 30308509 PMC6257099

[2] Ozsoy Z Demir E. Which bariatric procedure is the most popular in the world? A bibliometric comparison. Obes Surg. (2018) 28:2339–52. 10.1007/S11695-018-3163-6/TABLES/4 29512038

[3] Aiolfi A Tornese S Bonitta G Rausa E Micheletto G Bona D. Roux-en-Y Gastric Bypass: systematic review and Bayesian network meta-analysis comparing open, laparoscopic, and robotic approach. Surg Obes Relat Dis. (2019) 15:985–94. 10.1016/J.SOARD.2019.03.006 31104958

[4] Gagner M Gumbs AA Milone L Yung E Goldenberg L Pomp A. Laparoscopic sleeve gastrectomy for the super-super-obese (body mass index >60 kg/m(2)). Surg Today. (2008) 38:399–403. 10.1007/S00595-007-3645-Y 18560961

[5] Parikh MS Shen R Weiner M Siegel N Ren CJ. Laparoscopic bariatric surgery in super-obese patients (BMI > 50) is safe and effective: a review of 332 patients. Obes Surg. (2005) 15:858–63. 10.1381/0960892054222632 15978159

[6] Bindal V Sethi D Pandey D. Robotic primary bariatric surgery. Dig Med Res. (2021) 4:56–56. 10.21037/DMR-21-33

[7] Bauerle WB Mody P Estep A Stoltzfus J El Chaar M. Current trends in the utilization of a robotic approach in the field of bariatric surgery. Obes Surg. (2022) 33(2):482–91. 10.1007/S11695-022-06378-1 36572836 PMC9792156

[8] Cadiere GB Himpens J Vertruyen M Favretti F. The world’s first obesity surgery performed by a surgeon at a distance. Obes Surg. (1999) 9:206–9. 10.1381/096089299765553539 10340781

[9] Horgan S Vanuno D. Robots in laparoscopic surgery. J Laparoendosc Adv Surg Tech A. (2001) 11:415–9. 10.1089/10926420152761950 11814134

[10] Economopoulos KP Theocharidis V McKenzie TJ Sergentanis TN Psaltopoulou T. Robotic vs. Laparoscopic Roux-en-Y Gastric Bypass: a systematic review and meta-analysis. Obes Surg (2015) 25:2180–9. 10.1007/S11695-015-1870-9 26344797

[11] Li K Zou J Tang J Di J Han X Zhang P. Robotic versus laparoscopic bariatric surgery: a systematic review and meta-analysis. Obes Surg. (2016) 26:3031–44. 10.1007/S11695-016-2408-5 27726045

[12] Totaro A Campetella M Bientinesi R Gandi C Palermo G Russo A The new surgical robotic platform HUGO™ RAS: system description and docking settings for robot-assisted radical prostatectomy. Urologia. (2022) 89(4):603–9. 10.1177/03915603221107855 35765756

[13] Ragavan N Bharathkumar S Chirravur P Sankaran S Mottrie A. Evaluation of hugo RAS system in Major urologic surgery: our initial experience. J Endourol. (2022) 36(8):1029–35. 10.1089/END.2022.0015 35156838

[14] Sarchi L Mottaran A Bravi CA Paciotti M Farinha R Piazza P Robot-assisted radical prostatectomy feasibility and setting with the hugo™ robot-assisted surgery system. BJU Int. (2022) 130:671–5. 10.1111/BJU.15819 35689414

[15] Bravi CA Paciotti M Sarchi L Mottaran A Nocera L Farinha R Robot-assisted radical prostatectomy with the novel hugo robotic system: initial experience and optimal surgical set-up at a tertiary referral robotic center. Eur Urol. (2022) 82:233–7. 10.1016/J.EURURO.2022.04.029 35568597

[16] Bravi CA Paciotti M Balestrazzi E Piro A Piramide F Peraire M Outcomes of robot-assisted radical prostatectomy with the hugo RAS surgical system: initial experience at a high-volume robotic center. Eur Urol Focus. (2023) :S2405–4569. 10.1016/J.EUF.2023.01.008. [Online ahead of print] 36690548

[17] Alletti SG Chiantera V Arcuri G Gioè A Oliva R Monterossi G Introducing the new surgical robot HUGO™ RAS: system description and docking settings for gynecological surgery. Front Oncol. (2022) 12:eCollection. 10.3389/FONC.2022.898060 PMC9218341 35756633

[18] Monterossi G Pedone Anchora L Gueli Alletti S Fagotti A Fanfani G Scambia G. The first European gynaecological procedure with the new surgical robot Hugo™ RAS. A total hysterectomy and salpingo-oophorectomy in a woman affected by BRCA-1 mutation. Facts Views Vis Obgyn (2022) 14:91–4. 10.52054/FVVO.14.1.014 35373554 PMC9612853

[19] Raffaelli M Gallucci P Voloudakis N Pennestrì F de Cicco R Arcuri G The new robotic platform Hugo™ RAS for lateral transabdominal adrenalectomy: a first world report of a series of five cases. Updates Surg. (2023) 75(1):217–25. 10.1007/S13304-022-01410-6 36333563 PMC9834370

[20] Palmisano S Giuricin M Casagranda B de Manzini N. Zero frequency of internal hernias after laparoscopic double loop gastric bypass without closure of mesenteric defects. Surg Today. (2014) 44:1920–4. 10.1007/S00595-014-0916-2 24809335

[21] Pennestrì F Gallucci P Prioli F Giustacchini P Ciccoritti L Sessa L Barbed vs conventional sutures in bariatric surgery: a propensity score analysis from a high-volume center. Updates Surg. (2019) 71:113–20. 10.1007/S13304-018-0589-2 30191533

[22] Pennestrì F Prioli F Sessa L Gallucci P Ciccoritti L Giustacchini P Early routine upper gastrointestinal contrast study following bariatric surgery: an indispensable postoperative care or a medicolegal heritage? Obes Surg. (2019) 29:1995–8. 10.1007/S11695-019-03850-3 30945153

[23] Dindo D Demartines N Clavien PA. Classification of surgical complications: a new proposal with evaluation in a cohort of 6336 patients and results of a survey. Ann Surg. (2004) 240:205. 10.1097/01.SLA.0000133083.54934.AE 15273542 PMC1360123

[24] Pennestrì F Sessa L Prioli F Gallucci P Ciccoritti L Greco F Robotic vs laparoscopic approach for single anastomosis duodenal-ileal bypass with sleeve gastrectomy: a propensity score matching analysis. Updates Surg. (2023) 75(1):175–87. 10.1007/S13304-022-01381-8 36161395 PMC9834101

[25] Pennestrì F Sessa L Prioli F Salvi G Gallucci P Ciccoritti L Single anastomosis duodenal-ileal bypass with sleeve gastrectomy (SADI-S): experience from a high-bariatric volume center. Langenbecks Arch Surg. (2022) 407:1851–62. 10.1007/S00423-022-02501-Z 35352174 PMC9399205

[26] Fantola G Moroni E Runfola M Lai E Pintus S Gallucci P Controversial role of robot in primary and revisional bariatric surgery procedures: review of the literature and personal experience. Front Surg. (2022) 9. 10.3389/FSURG.2022.916652 35711697 PMC9194091

[27] Larkins KM Mohan HM Gray M Costello DM Costello AJ Heriot AG Transferability of robotic console skills by early robotic surgeons: a multi-platform crossover trial of simulation training. J Robot Surg. (2022). 10.1007/S11701-022-01475-W. [Online ahead of print] PMC10209232 36324049

[28] Fersahoğlu MM Ergin A Çiyiltepe H Fersahoglu AT Bulut NE Bilgili AC Comparison of the pretzelflex retractor and nathanson retractor in laparoscopic sleeve gastrectomy surgery. Obes Surg. (2021) 31:4963–9. 10.1007/S11695-021-05680-8 34436716

[29] Hatao F Imamura K Ishibashi Y Kawasaki K Yamazaki R Morita Y. Liver retraction using an L-shaped retractor during sleeve gastrectomy. Surg Today. (2022) 52:574–9. 10.1007/S00595-021-02430-2 34853882

[30] Gojayev A. Yüksel C Mercan Ü Çaparlar MA Cetindag O Akbulut S The effect and clinical significance of using nathanson liver retractor on liver function tests in laparoscopic gastric cancer surgery. Pol Przegl Chir. (2021) 94:54–61. 10.5604/01.3001.0015.3544 35195072

[31] Hiramatsu K Aoba T Kamiya T Mohri K Kato T. Novel use of the nathanson liver retractor to prevent postoperative transient liver dysfunction during laparoscopic gastrectomy. Asian J Endosc Surg. (2020) 13:293–300. 10.1111/ASES.12735 31389200 PMC7379723

[32] Garofalo F Mongelli F Cristaudi A la Regina D Podetta M Marengo M Laparoscopic gastric bypass conversion to SADI-S with use of indocyanine green fluoroscopy. Obes Surg. (2022) 32:2823–4. 10.1007/S11695-022-05929-W 35697994

[33] Balla A Corallino D Quaresima S Palmieri L Meoli F Cordova Herencia I Indocyanine green fluorescence angiography during laparoscopic bariatric surgery: a pilot study. Front Surg. (2022) 9:906133. 10.3389/FSURG.2022.906133 35693301 PMC9178117

[34] Pavone G Fersini A Pacilli M Cianci P Ambrosi A Tartaglia N. Anastomotic leak test using indocyanine green during laparoscopic Roux-en-Y Gastric Bypass: a cohort study. Ann Med Surg (2022) 84:104939:eCollection. 10.1016/J.AMSU.2022.104939 PMC9758372 36536736

[35] Jiang HP Lin LL Jiang X Qiao HQ. Meta-analysis of hand-sewn versus mechanical gastrojejunal anastomosis during laparoscopic Roux-en-Y Gastric Bypass for morbid obesity. Int J Surg (2016) 32:150–7. 10.1016/J.IJSU.2016.04.024 27107663

[36] Awad S Aguilo R Agrawal S Ahmed J. Outcomes of linear-stapled versus hand-sewn gastrojejunal anastomosis in laparoscopic Roux en-Y Gastric Bypass. Surg Endosc (2015) 29:2278–83. 10.1007/S00464-014-3942-7 25380709

[37] Wesley Vosburg R Haque O Roth E Robotic VS. Laparoscopic metabolic and bariatric surgery, outcomes over 5 years in nearly 800,000 patients. Obes Surg (2022) 32:2341–8. 10.1007/S11695-022-06082-0 35499639

